# Supplementary material for: Novel Driver Strength Index highlights important cancer genes in TCGA PanCanAtlas patients
Source: PeerJ. 2022 Aug 11;10:e13860. doi: 10.7717/peerj.13860 (PMC9375969; doi:10.7717/peerj.13860)
Supplement: Supplemental Information 2 [file peerj-10-13860-s002.zip › COHORTS/genes plots/detailed/2021_8_16_14_9_distribution_events_detailed_35.pdf]

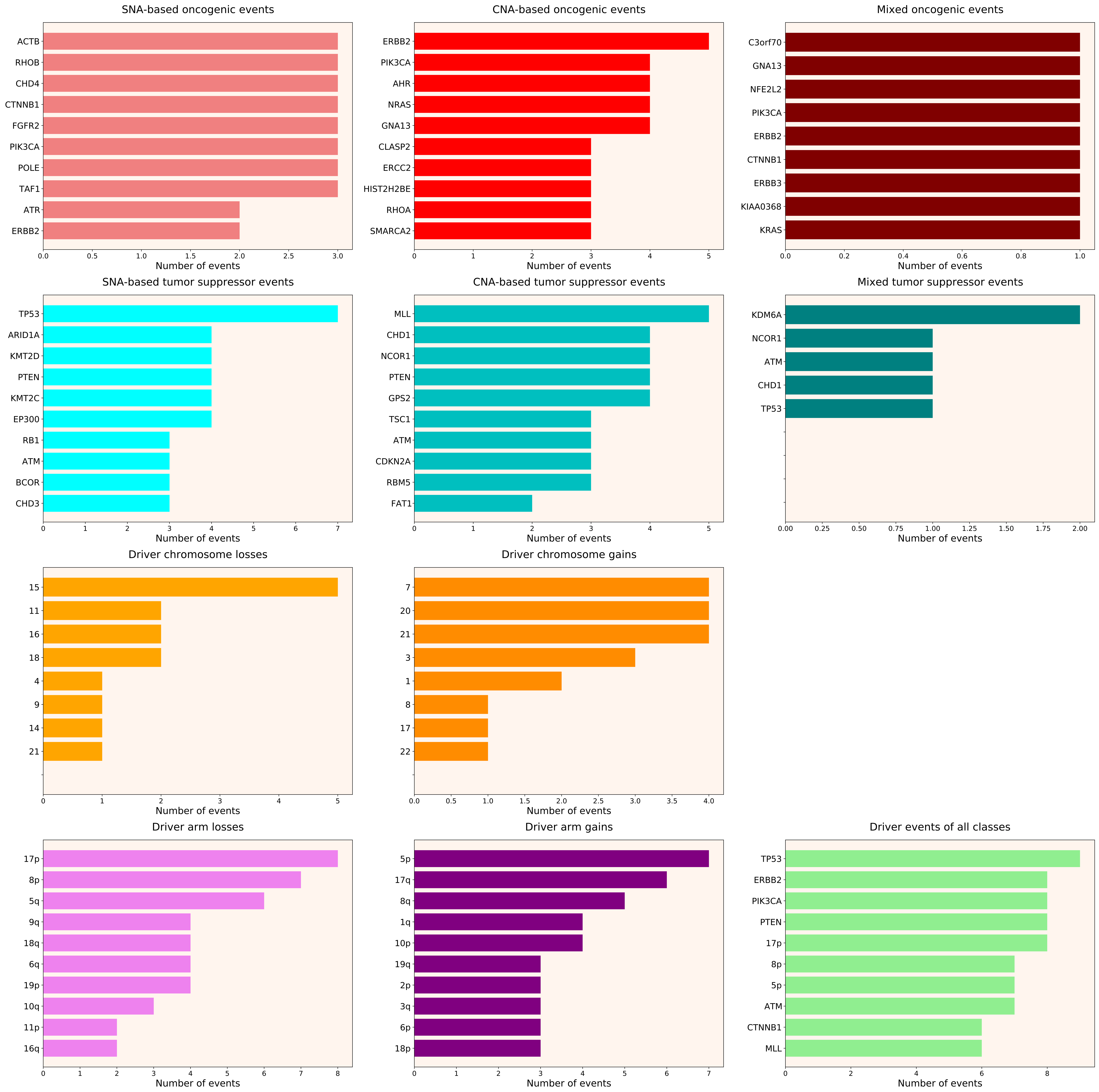
SNA-based oncogenic events
ACTB
RHOB
CHD4
CTNNB1
FGFR2
PIK3CA
POLE
TAF1
ATR
ERBB2
0.0 0.5 1.0 1.5 2.0 2.5 3.0
Number of events
CNA-based oncogenic events
ERBB2
PIK3CA
AHR
NRAS
GNA13
CLASP2
ERCC2
HIST2H2BE
RHOA
SMARCA2
0 1 2 3 4 5
Number of events
Mixed oncogenic events
C3orf70
GNA13
NFE2L2
PIK3CA
ERBB2
CTNNB1
ERBB3
KIAA0368
KRAS
0.0 0.2 0.4 0.6 0.8 1.0
Number of events
SNA-based tumor suppressor events
TP53
ARID1A
KMT2D
PTEN
KMT2C
EP300
RB1
ATM
BCOR
CHD3
0 1 2 3 4 5 6 7
Number of events
CNA-based tumor suppressor events
MLL
CHD1
NCOR1
PTEN
GPS2
TSC1
ATM
CDKN2A
RBM5
FAT1
0 1 2 3 4 5
Number of events
Mixed tumor suppressor events
KDM6A
NCOR1
ATM
CHD1
TP53
0.00 0.25 0.50 0.75 1.00 1.25 1.50 1.75 2.00
Number of events
Driver chromosome losses
15
11
16
18
4
9
14
21
0 1 2 3 4 5
Number of events
Driver chromosome gains
7
20
21
3
1
8
17
22
0.0 0.5 1.0 1.5 2.0 2.5 3.0 3.5 4.0
Number of events
Driver arm losses
17p
8p
5q
9q
18q
6q
19p
10q
11p
16q
0 1 2 3 4 5 6 7 8
Number of events
Driver arm gains
5p
17q
8q
1q
10p
19q
2p
3q
6p
18p
0 1 2 3 4 5 6 7
Number of events
Driver events of all classes
TP53
ERBB2
PIK3CA
PTEN
17p
8p
5p
ATM
CTNNB1
MLL
0 2 4 6 8
Number of events
